# Effects of Serial Multiple Exemplar Training on Bidirectional Naming in Children with Autism

**DOI:** 10.1007/s40616-024-00203-9

**Published:** 2024-02-23

**Authors:** Roy Salomonsen, Sigmund Eldevik

**Affiliations:** 1 Habilitation Center for Children and Adolescents, University Hospital of North Norway, Tromsø, Norway; 2 Department of Behavioral Science, Oslo Metropolitan University, Oslo, Norway

**Keywords:** Bidirectional naming, Autism, Serial multiple exemplar training, Stimulus generalization

## Abstract

This study examined the effect of a serial multiple exemplar training (S-MET) procedure on bidirectional naming (BiN) in four preschool children diagnosed with autism spectrum disorder (ASD). A non-concurrent multiple baseline design was used to evaluate the effects of training listener and speaker behavior for one stimulus at a time until BiN occurred. When BiN occurred, probes were conducted to measure whether generalization occurred across settings and people. Three out of four participants’ responding met the mastery criterion for BiN, while the fourth participant improved her performance. The results of this study suggest that S-MET may be a promising intervention and contribute to our knowledge about learning histories required for BiN.

*Bidirectional Naming* (BiN) is defined as a “Higher-order operant involving a bidirectional relation between speaker and listener behaviors. The teaching of one of these components suffices to establish both” (Miguel, 2016, p. 134). This bidirectional relationship implies that, when you teach an individual to respond to a stimulus as a listener (e.g., pointing to butterfly in response to “Point to butterfly”), they will respond as a speaker to the same stimulus without direct training (e.g., saying “butterfly” in the presence of a butterfly), and vice versa. BiN may also emerge by just presenting a novel stimulus to the individual and naming it, and, as a result of merely observing this episode, both the listener and the speaker behavior emerges (Miguel, 2016; Carnerero & Pérez-González, 2014).

When Horne and Lowe (1996) introduced their naming hypothesis, they described how naming is established in everyday interactions between parents and typically developing children. *Listener responses* are usually first evoked when parents make requests for (“give me the…) or point out objects and then reinforce the child’s listener responses (e.g., looking at, grabbing). Later, *echoic behavior* is established when parents reinforce the child’s attempts to repeat their vocalizations or by inadvertently reinforcing the child’s echoic behavior accompanying the relevant listener behavior (e.g., saying “You did it!” when the child touches the ball and says “ba” when asked to “find the ball”). Horne and Lowe (1996) emphasized the essential role of echoic behavior in the naming relation. An important effect of the echoic is that it “allows listener relations to expand into speaker relations” (Olaff & Holth, 2020, p. 2). After many such instances, the child’s vocalizations will eventually come under the control of non-verbal stimuli in the child’s environment in the form of *tacts*. When the child starts echoing the parents’ tacts, the echoing will evoke listener behavior in response to the child’s own speaker behavior. Miguel (2016) introduced the term *Bidirectional Naming*, which highlights this integration of the listener and speaker repertoires within the individual. The child starts to respond to their own speaker behavior as an effective listener (e.g., orient to the ball when they hear themselves say “ball”), and vice versa. At this point, the child “starts to listen with understanding” (Miguel, 2016, p. 128).

At around 2 to 3 years old, children demonstrate a rapid acquisition of verbal behavior, commonly known as the *vocabulary spurt* (Labrell et al., 2014). Some researchers have suggested that BiN may explain this “vocabulary spurt” (Greer & Longano, 2010). The generative nature of BiN, which involves learning new words with less exposure to direct contingencies of reinforcement, may be necessary for the exponentially expanding vocabulary (Greer & Ross, 2008). For many children with ASD and other developmental delays, BiN does not develop, and results in verbal behavior training becoming time consuming, always requiring the direct training of both listener and speaker relations (LaFrance & Miguel, 2014). Establishing BiN could enable children to learn many more words and teachers to focus on more advanced language skills, resulting in reduced instruction hours. Additionally, it has been suggested that BiN is a prerequisite for several important skills, such as writing, spelling, and reading (Greer & Ross, 2008), observational learning (Gilic & Greer, 2011), and stimulus categorization (e.g., sorting pictures of physical dissimilar stimuli into categories such as fruit, toys etc.; Lee et al., 2015; Lowe et al., 2005; Miguel et al., 2008). Multiple exemplar instruction is one procedure that has been demonstrated to establish BiN.

*Multiple Exemplar Instruction (MEI)* involves the simultaneous rotation of both the training stimuli and operants in each trial, and also training using sets of multiple stimuli before probing for emergent responding. An example of MEI with five stimuli would involve presenting a listener trial (“point to baboon”), followed by an impure tact trial (shown a picture of a heron and asked, “what is it?”), followed by a matching trial (“match donkey”) and a pure tact trial of wasp (shown a picture of wasp), followed by a listener trial (“point to tern”), and so on until all the responses classes were targeted with all the stimuli in the set, and responding across response classes reached a mastery criterion. Sometimes, if BiN is not established after training with one set of stimuli, then the sequence of MEI and probing of BiN using new sets of stimuli may be repeated until BiN is established (see Olaff et al., 2017).

MEI has been demonstrated to establish BiN in children with and without developmental delays (e.g., Greer et al., 2005; Gilic & Greer, 2011; Fiorile & Greer, 2007). Using MEI to establish BiN involves training the listener and speaker repertoires (LaFrance & Tarbox, 2020). Greer et al. (2005) utilized an MEI procedure to establish the speaker component of BiN in three preschool children with developmental delays. The stimuli used in the study were divided into three sets for probing and MEI. Each set consisted of five stimuli. The first step was a pretest probe of set one for BiN. They taught the participants to match each stimulus to a sample to mastery, while the teacher simultaneously said the name of the stimulus. After teaching matching-to-sample, each stimulus was probed for listener responding (pointing), and speaker responding, *pure* tact (naming a stimulus in response to being shown a stimulus), and *impure* tact (naming a stimulus in response to being shown a stimulus and asked what it is). The third step was the MEI condition which involved teaching the five stimuli in set two across four responses classes. The response classes were two listener responses (matching-to-sample while the teacher said the name of the stimulus and pointing) and two speaker responses (pure tact and impure tact). During each trial, the experimenter rotated the stimulus and the responses until every stimulus was mastered across the four response classes. To demonstrate BiN, new sets of untrained stimuli were probed in the same way as they were during the pretest probes. BiN probes were conducted on set one and three to check whether the speaker part of BiN had emerged. A participant failed to demonstrate BiN if their speaker responding did not reach mastery for sets of new stimuli. The results showed that after MEI, all three participants demonstrated BiN.

Greer et al. (2007) compared MEI to single exemplar instruction (SEI) in children with autism. SEI did not involve rotating responses, but instead involved training each operant involved in BiN separately to mastery. An example of SEI was training only the listener responses for a set of stimuli (e.g., baboon, heron, donkey, wasp, tern). When listener responses for all stimuli had been trained to mastery, participants were taught to tact the same stimuli in separate sessions. Training continued until responding for all stimuli had been trained for all targeted operants. The results demonstrated that only MEI resulted in BiN.

In some MEI experiments, 10–13 steps or phases were required to establish BiN (see Hawkins et al., 2009; Olaff & Holth, 2020). MEI can be complex and time consuming to implement. In an effort to reduce the resource intensive nature of MEI and optimize procedures that establish emergent responding, instead of training whole sets of exemplars concurrently and probing other sets for generalized responding, it may be just as effective to train exemplars serially – one exemplar at a time; otherwise referred to as serial multiple exemplar training (S-MET; Schnell et al., 2018). White et al. (1998) described this process as follows: “If training on one does not produce generalization, training is directed to another exemplar, and so on, until generalization to other exemplars is noted” (p. 28). S-MET could have some potential advantages compared to MEI in that it provides detailed information about when generalized responding occurs, and can create more efficiency in teaching due to the fact that more sets of stimuli than necessary are not targeted. It is important to note that MEI is not the same as multiple exemplar training (MET). MET consists of training different stimulus or response exemplars *within an operant class*, usually until generalization to untrained stimuli (or responses topographies) occur. An example of this would be to train an individual to tact three different looking cars (e.g., a blue Toyota SUV, a white Honda, and a red toy car), and then probe to see if the individual is able to tact exemplar number four and five (two cars that look different from the first three and from one another) without training. MEI, on the other hand, “consists of the rotation of *different verbal operants with different functions* (e.g., match, point to, tact, multiply controlled tact) across a series of consecutive trials” (LaFrance & Tarbox, 2020, p. 13).

S-MET has been successfully used to teach children with autism answers to wh-questions (Jahr, 2001), to initiate and sustain cooperative play (Jahr et al., 2000), to ask for missing items (Wójcik et al., 2020), and to teach children with a phonological disorder to articulate sounds or sound combinations during vocal imitation training (Eikeseth & Nesset, 2003). Eldevik et al. (2016) used S-MET to promote the transfer of correct use of past tense verbs to untrained verbs among four participants with autism, aged 6 to 20. Prompt fading and shaping were used to teach one verb at a time. When one verb was trained to mastery, the next verb from a list of untrained verbs was probed for the correct verb form. If the participant’s responding during the probe did not reach mastery, the verb was directly trained to mastery. If the participant responded correctly, the next untrained verb was probed. This continued until the participants responded correctly on five consecutive probes. S-MET resulted in all four participants emitting the correct verb form when presented with new untrained verbs. The participants required training with eight to 13 verbs before generalized responding was observed.

Both MEI and MET were implemented in the current study. As is done when implementing MEI, different operants (listener and speaker responses) were trained for a particular stimulus before the next one was introduced. However, listener and speaker responses were not rotated across trials as is commonly done when conducting MEI. The responses were trained separately to mastery, as is commonly done when conducting MET. The purpose of the present study was to examine whether serial multiple exemplar training (S-MET) would establish BiN in preschool children diagnosed with autism. To our knowledge, no study has evaluated the effect of S-MET on establishing BiN in children. Continuous probing for BiN helped in determining whether and when generalized responding occurred. Probing and training one exemplar at a time provides data that may enable a more fine-grained analysis of the development of BiN.

## Method

### Participants

Four children, Dave, Elliot, Isaac, and Eva, participated in the study. The participants were between 3 and 4 years of age, and they had all been diagnosed with autism or pervasive developmental disorder based on the ICD-10 criteria (World Health Organization, 2007). All of the participants were diagnosed using the Autism Diagnostic Observation Schedule-Second Edition (ADOS-2; Lord et al., 2012), and the Autism Diagnostic Interview-Revised (ADI-R; Rutter et al., 2003). They were enrolled in early intensive behavioral intervention (EIBI) programs in local day care centers. None of the participants had received direct training in BiN skills prior to this study. At the time of the study, all of the participants were assessed using the Assessment of Basic Learning and Language Skills-Revised (ABLLS-R; Partington, 2006). See Table 1 for a more detailed description of the participants’ mastery of each skill area.

**Table 1:** Participant characteristics

Characteristics	Dave	Elliot	Isac	Eva
Gender	Male	Male	Male	Female
Age in months	49	56	49	43
Diagnoses	Autism^a^	Autism^a^	Autism^a^	PDD-NOS^b^
Months in EIBI^c^	8	11	10	10
Weekly hours of EIBI	22	20	20	5–15
ABLLS–R scores^d^				
Receptive language (C)	89%	93%	73%	60%
Vocal imitation (E)	93%	95%	88%	75%
Labeling (G)	61%	67%	44%	35%
Total of C, E, and G	78%	83%	64%	53%
Total of all 25 skill areas	72%	76%	53%	40%

^a^Childhood autism

^b^Pervasive developmental disorder, unspecified

^c^Early Intensive Behavioral Intervention

^d^The ABLLS–R scores indicates the participant’s mastery of each skill area in the Assessment of Basic Language and Learning Skills shown in percent of maximum possible scores

To be eligible for the study, the participants had to vocally imitate (echo) sentences of up to 3 to 4 words. Participants’ ABLLS-R scores were used to determine whether they had the minimum skills required to be included in the study. They had to respond as a listener to at least 50 words, and also tact at least 50 words. Listener responding was defined as the participants touching or pointing to the correct stimulus (picture) from an array of stimuli when asked. Tacting was defined as the participants emitting the correct name of the stimulus when asked. The participants also could not engage in BiN to qualify for the study. BiN for a particular stimulus was defined as the participants showing correct listener and speaker behavior after observing another person naming a stimulus twice. If the participants demonstrated BiN for more than two out of the five probes, they were excluded from the study.

### Setting

The study was conducted in the participants’ local day care centers, where they received their daytime EIBI-programming. Probing and intervention sessions were conducted in separate teaching rooms. These were the same rooms that were used for most of their other intervention programs. The participant and the experimenter were seated facing each other at a table. The sessions lasted around 15 to 30 minutes. The first author trained and supervised the day care center teachers who, together with the first author, implemented the intervention. Generalization was assessed in two ways. The first was to evaluate generalization across activities not related to training sessions (e.g., meals, free play, and during transitions), and across settings (e.g., play areas, bathrooms, locker rooms, etc.). The second was to assess generalization across people who were not involved in the project (e.g., staff, parents, or children attending the day care centers). These second generalization assessments were conducted in the participants’ teaching room.

### Materials

We recorded data using data sheets tailored for the project. They were the same for every participant. Written descriptions of the probe and intervention procedures were available for the teachers, both in the teaching rooms and in the generalization settings. Potential reinforcers, stimuli such as toys, food, or games, were provided through a token economy system, tailored to each participant. The token system was the same one used during their EIBI sessions. Stimuli that were used as backup reinforcers were chosen based on teacher and parent reports. The reinforcement schedule for the token economy was a continuous reinforcement schedule (CFR) during initial training trials of newly introduced stimuli. After the mastery criterion was met, the schedule was thinned to a variable ratio 3 schedule (VR3).

Pictures cards were prepared to assess and teach BiN. They were 9 cm by 9 cm color photos of stimuli against a transparent/white background. For each participant, we chose stimuli from one of the following categories: wild animals, fruit and vegetables, or children’s characters from TV/movies, etc. The participants had some knowledge of some members of these categories; more uncommon members of the category were therefore selected*.* Dave’s cards included children’s characters from TV-series such as Bart from The Simpsons and Dizzy from Bob the Builder, from children’s movies such as Dumbo, Simba, and Pinocchio, or game characters like Mario. Elliot’s cards included fruit, berries, and vegetables (e. g., ginger, chili, artichoke, parsnips, raspberry, and grapefruit). Isaac and Eva’s cards were wild animals (e.g., a gorilla, wasp, scorpion, llama, hedgehog, or pelican). Stimuli used for generalization probes were conducted using objects found in the daycare centers that the participants typically did not interact with, such as knife steel, screw bit, file, egg slicer, wrench, and corkscrew. For Dave’s generalization probes, pictures of children’s characters were used.

### Dependent variable and data collection

The primary dependent variable was the percentage of correct responding on probes for BiN. A probe for BiN for one stimulus consisted of eight listener trials and five speaker trials. All listener and speaker trials had to be correct for the BiN probe to be scored as correct. When probing for listener responding there was a possibility that the participant could respond correctly by chance. This was not the case when probing for speaker responding, which is the reason that there were more listener (8) trials compared to the speaker trials (5). The BiN probe data were calculated as a percentage of correct responses. A correct listener response was defined as touching/showing/selecting the correct stimulus from an array of five stimuli after being given the instruction, “Touch (stimulus).” A correct response was recorded if the participant correctly touched/showed/selected the stimulus within five seconds of the instruction. An incorrect response was recorded if the participant did not respond within five seconds, or selected an incorrect stimulus. A correct speaker response was defined as a correct tact of the stimulus presented when asked, “What is it?”. A correct speaker response was recorded if the participants correctly labeled the stimulus within five seconds of the instruction. An incorrect response was recorded if the participant did not respond within five seconds, or labeled the stimulus incorrectly. We recorded data as correct or incorrect on a trial-by-trial basis. During generalization probes, the dependent variable was the percentage of correct speaker responses. A response was recorded as correct if the participant labeled the stimulus within five seconds when asked, “What is it?”. A response was recorded as incorrect if the target response was not emitted within five seconds of the question, or it was labeled incorrectly. Probing only the speaker part of BiN was done for practical reasons. The reasoning was that it would be easier to probe speaker responses during ongoing activities during the course of a typical day at school or during sessions. Whereas probing listener responses would require an interruption in the activity and involve special arrangements of the stimuli.

### Design

We used a non-concurrent multiple baseline design (Watson & Workman, 1981) across participants to evaluate the effects of the S-MET procedure. The experimental sequence was initiated at different times for each participant, and we randomly assigned the participants to different baseline lengths. Dave was assigned to a one-week baseline length, Elliot to a three-week baseline, Isaac to a four-week baseline, and Eva to a five-week baseline. For the one-week condition, there were two baseline probes, on day 1 and day 7. For the three-week condition, there were three probes, on day 1, day 10, and day 21. The four-week condition had three baseline probes, on day 1, day 13, and day 28. Lastly, for the five-week condition, there were three baseline probes, on day 1, day 20, and day 35.

### Procedure

During pre-experimental procedures and BiN probes, participants only received reinforcement for general effort and attention. We provided praise (e.g., “You are working so hard!”) and a token when the participant was looking at the experimenter or sitting nicely. During S-MET, we provided reinforcement (token and praise; e.g., “Baboon, that is correct”) contingent on correct responding to the target stimulus. The consequence for prompted correct responses was praise, only. Reinforcement was not provided for incorrect or no responding. The order of the experimental procedure is displayed in Table 2.

**Table 2:** The order of the experimental conditions

Steps	Description
1	Pre-experimental procedures to ensure that the child could imitate the name of the stimuli, but not respond as a listener or speaker.
2	Baseline probes. Each baseline session consisted of BiN tests of 5 stimuli.
3	Generalization probes conducted prior to S-MET to test the speaker part of BiN in other settings and with other people.
4	Serial multiple exemplar training (S-MET). Each new stimulus starts with a BiN probe. Only response classes, either the listener part and/or the speaker part, that were not mastered were trained.
5	Generalization probes conducted after S-MET to test the speaker part of BiN in other settings and with other people.

#### Pre-experimental assessments

Pre-experimental assessments included an echoic, tact, and listener assessment for every stimulus and they were conducted before the baseline condition. The stimuli were probed in random order for each of the three assessments. The participants were given two opportunities to respond per stimulus. The participants had to respond correctly within five seconds of the instruction. *An echoic assessment* was administered to see whether the participants were able to echo the name of the stimulus. We presented the instruction “say (stimulus).” Stimuli that the participants were unable to echo were excluded from the study. *A tact assessment* was administered to determine whether the participants could respond as a speaker. We showed the participants one stimulus at a time and asked, “What is it?”. If the participants could tact the stimulus (responded correctly during one of two opportunities), the stimulus was excluded from the study. *A listener assessment* involved presenting the target stimulus along with two stimuli that were known and two stimuli that were unknown in a line in front of the participant, for a total of five stimuli. We instructed the participants to touch the target stimulus (e. g., “touch garlic”). Trials with the target stimulus were randomly interspersed with trials with the two known stimuli. If the participants responded correctly during both trials of the target stimulus, the stimulus was excluded from the study. Stimuli that were not excluded in the pre-experimental assessment were randomly assigned to either the baseline or the S-MET condition. Praise (e.g., “I like how hard you are working”) and tokens were provided contingent on looking at the experimenter, on good sitting, and on responding, and distributed equally between correct and incorrect responses. We provided praise on a CRF schedule, and tokens on a VR3 schedule. One to three assessment sessions were conducted per day. Sessions were conducted two to five days each week.

#### Baseline probes

Each baseline session consisted of five probes with five unknown stimuli. The BiN probes for each stimulus were conducted in three steps:

##### Step 1 was modeling of the tact

We showed a picture of the target stimulus while the participants observed, and then said the name of the stimulus twice, with five seconds in between each presentation of the stimulus name. In step 1, the only requirements for the participants were to look at the experimenter and the stimulus presented. This step could be considered to be an approximation of how children with BiN skills learn the names of novel stimuli in natural settings.

##### Step 2 was the listener probe

We placed five stimuli in a line on the table in front of the participants. These included the target stimulus, two known, and two unknown stimuli. We instructed the participants to touch a stimulus (e.g., “touch elephant”). A total of eight trials were conducted; asking for the target stimulus in three trials, and the two known stimuli in five trials. In order to pass the listener probe, the participants were required to respond correctly during all eight trials. The order of the stimuli was randomized across trials, while the positions on the table were also rotated. The participants were not instructed to touch the two unknown stimuli. The unknown stimuli were later probed and trained using S-MET.

##### Step 3 was the speaker probe

We showed the target stimulus to the participants and asked, “What is it?” A total of five trials were conducted and it involved asking the participants to tact the target stimulus in three trials and two known stimuli in two trials. In order to pass the speaker probe, the participants were required to respond correctly during all five trials.

To pass the BiN probe, the participants’ responding needed to meet the above criteria during both the listener and speaker probes. Hence, a total of 13 (eight + five) consecutive correct trials of listener and speaker responses for each stimulus probed were required. A total of 65 trials were conducted per baseline session. We only presented the tact during step 1 and not during steps 2 and 3.

#### Pre-training generalization probes

Pre-training generalization probes were conducted on the same day as or the day after the last baseline probe session. Two types of generalization probes were conducted. During the *settings probe,* we assessed responding to three stimuli in other settings and using different activities, but with the experimenters. During the *people probe*, the assessment was conducted in the participants’ regular teaching room but by people other than the experimenters (e.g., children attending the day care center, staff, or the participant’s mother or father visiting the day care center). Staff who were part of the project were present during these generalization probes, but did they not conduct the probe trials. The novel person (e.g., the father) showed the participant the stimulus while saying the name of it once (e.g., held up the wrench and said “wrench”). After 30 seconds, the novel person showed the participant the stimulus again and asked, “What is it?”. The participants had one opportunity to answer correctly. Praise was provided for responding to the instruction, whether or not the responses were correct. No stimuli were probed twice. The generalization probes differed from the BiN probes used in baseline and S-MET in three ways: (1) unknown objects found in the participant’s environment were utilized, and not stimuli from the categories used in baseline and S-MET, (2) the name of each stimulus was only presented once, and (3) only the speaker behavior was assessed. We conducted generalization probes in this manner because this more closely approximates how children learn language in the natural environment. For Dave, generalization probes were not conducted prior to the intervention since he was the first participant in this study to complete the procedures. We introduced pre-training probes for the remaining participants in order to more effectively evaluate the results of the post-training probes.

#### Serial Multiple Exemplar Training (S-MET)

S-MET was conducted on the same day as or the day after the pre-training generalization probes. Stimuli for S-MET were randomly selected from stimuli not excluded during the pre-experimental assessment. We introduced training for one stimulus at a time. The intervention started with a BiN probe for the first stimulus. The probe was procedurally identical to baseline probes. If the participants mastered the BiN probe for the particular stimulus (e.g., baboon), the next stimulus on the list was probed for BiN (e.g., otter). If the participants failed the BiN probe of the stimulus (e.g., baboon), only the response forms of the target behavior the participant failed (listener response, speaker response, or both) were trained (e.g., if they failed the listener portion but passed the speaker portion, only the listener response was targeted). After we trained the target response to mastery, the next untrained stimulus (e.g., otter) became the target stimulus and was probed for BiN. Following this procedure, the remaining stimuli on the list were probed one at a time. We used the results of this probing procedure as one measure of evidence of BiN. After the participants engaged in BiN to mastery for three consecutive stimuli (e.g., otter, antelope, and condor), the training was concluded. For two of the participants, Elliot and Isaac, we established a higher criterion of five consecutive BiN probes to see whether this higher criterion would improve stimulus generalization.

If the participant’s responding did not meet mastery during the listener probe, we trained listener responding for that particular stimulus. Since listener training included echoic training, it will be described as *listener and echoic response training*, hereafter. During *listener and echoic response training*, we placed the target stimulus in a line on the table together with two known and two unknown stimuli. We presented the instruction “touch (stimulus).” We used a prompting procedure on the following trials if the participants responded incorrectly or did not respond to the instruction. The choice of prompts depended on which type of error the participants made. If the participants did not echo the instruction, an echoic prompt was used. We used a constant time delay of four seconds to fade the echoic prompt. For some participants, we modeled both the sequence of the echoic response and the pointing response if they did not select the stimulus. If needed, we physically blocked the participants’ hands by gently holding the them back so that they echoed the name before touching it. Another prompt involved positioning the correct stimulus closer to the participants to teach them to respond correctly as a listener. Position prompts were faded using a most-to-least procedure, fading the position prompt from full position, to half position, then to no position. An example of a prompt fade is “touch (stimulus),” and immediately providing the prompt “say (stimulus),” while blocking and then releasing the participants hands so that they could point to the target stimulus while the target stimulus was in full position. The echoic prompt was faded first, then the blocking prompt, and lastly the position prompt. During listener probing and training trials, the two unknown stimuli on the table were the next two stimuli on the list to be probed. For example, if the list of stimuli to be probed and trained consisted of baboon, bison, and octopus, the first to be probed was baboon, while the two unknowns would be bison and octopus. The known stimuli on the table were the last two mastered stimuli from probing and training. If no stimuli had been mastered at the start of the intervention or in baseline probes, stimuli were used that were mastered from the pre-experimental assessment or stimuli that we knew the participant was familiar with. If the participants responded incorrectly in a trial with previously mastered stimuli (distractors), they were trained using the same prompting and prompt fading procedures.

If the participant failed the speaker probe, we trained speaker responding. We held up the card, asked the question, “What is it?”, and prompted responding by modeling the correct response, “say (stimulus).” After a prompted trial, we repeated the instruction and then faded the prompt using a constant time delay of four seconds between the instruction and the echoic prompt. This was done until the participants responded independently to the instruction. The known stimuli we used in speaker training were the two previously mastered stimuli from probing and training. Previously mastered stimuli that the participants did not respond to correctly during training trials were trained, as was done during listener training. We used differential reinforcement during both listener and echoic response training, and speaker training. Correct prompted responses produced praise. Correct independent responses produced praise using a CRF schedule, and a token was delivered according to the reinforcement schedule that was in place (CRF or VR3). Incorrect responses did not produce praise or a token, and the participants only received corrective feedback (e.g., “No, it is not falcon. It is bluebird”).

The mastery criterion in listener and echoic training was eight consecutive correct trials in a random mix with mastered stimuli, provided that the target stimulus was asked for three times in this mix. We rotated the positions of the stimuli for each trial. In the speaker training, the criterion was five consecutive correct trials in a random mix with mastered stimuli, provided that the target stimulus was asked for three times in the mix. We conducted training between three to five days a week, for one to three sessions per day. Training was stopped for a few days if the participants or experimenters were ill. Each training session consisted of the probing and/or training of two to four stimuli. A varying number of training trials were targeted per session. An estimate based on the available data suggests that between 20 to 60 trials were conducted for each stimulus if both listener/echoic and speaker behavior were trained for the stimulus, fewer if only one response form was trained, and no trials if the participants demonstrated responding to mastery during the probe trials. We provided the participants short breaks during training sessions. Breaks in S-MET were not provided during BiN probes (steps 1–3), only during listener and echoic, and/or speaker training. An outline of the intervention procedure is displayed in Table 3.

**Table 3:** The procedure for serial multiple exemplar training. After assessing for BiN, different responses were trained depending on the results

Step	Item	Naming experience	BiN tests	If incorrect on listener test	If incorrect on speaker test	If incorrect on both tests	If correct on both tests
1	Dog	Experimenter named dog twice	First, the listener part of dog, then the speaker part was tested	The listener part of dog was trained to mastery, then the next stimulus was probed	The speaker part of dog was trained to mastery, then the next stimulus was probed	Both the listener part and speaker part of dog were trained to mastery, then the next stimulus was probed	The next stimulus was probed
2	Cat	Experimenter named cat twice	First, the listener part of cat, then the speaker part was tested	The listener part of cat was trained to mastery, then the next stimulus was probed	The speaker part of cat was trained to mastery, then the next stimulus was probed	Both the listener part and speaker part of cat were trained to mastery, then the next stimulus was probed	The next stimulus was probed
	The procedure continued until participants responded correctly to both listening responses and speaking responses in 3 consecutive BiN probes.						

#### Post-training generalization probes

For Dave, generalization probes were conducted as regular BiN probes similar to those conducted in the baseline and treatment conditions, which involved probing both listener and speaker responding. Full BiN probes were extensive and involved an abundance of probing material. Therefore, we simplified the generalization probes for the remaining participants. For Elliot, Isaac, and Eva, the generalization probes were procedurally identical to the pre-training generalization probes. Post-training generalization probes were conducted on the same day as the mastery criterion for BiN was reached for Dave, Elliot, and Eva, and on the following day for Isaac.

### Interobserver agreement

A subset of sessions had an independent observer present to collect data for the purpose of measuring interobserver agreement. Both observers independently scored the participant’s responses as either incorrect or correct. We calculated interobserver agreement by dividing the number of agreements by the number of disagreements plus agreements and multiplying by 100. IOA was assessed during 73.4% of Dave’s trials, 27.5% of Elliot’s trials, 31.7% of Isaac's trials, and 34.7% of Eva’s trials. The mean agreement was 98.7% for Dave (range 97.3–100%), 99.3% for Elliot (range 97.4–100%), 97.1% for Isaac (range 96.8–100%), and 95.3% for Eva (range 90.7–100%).

## Results

**Fig. 1:**
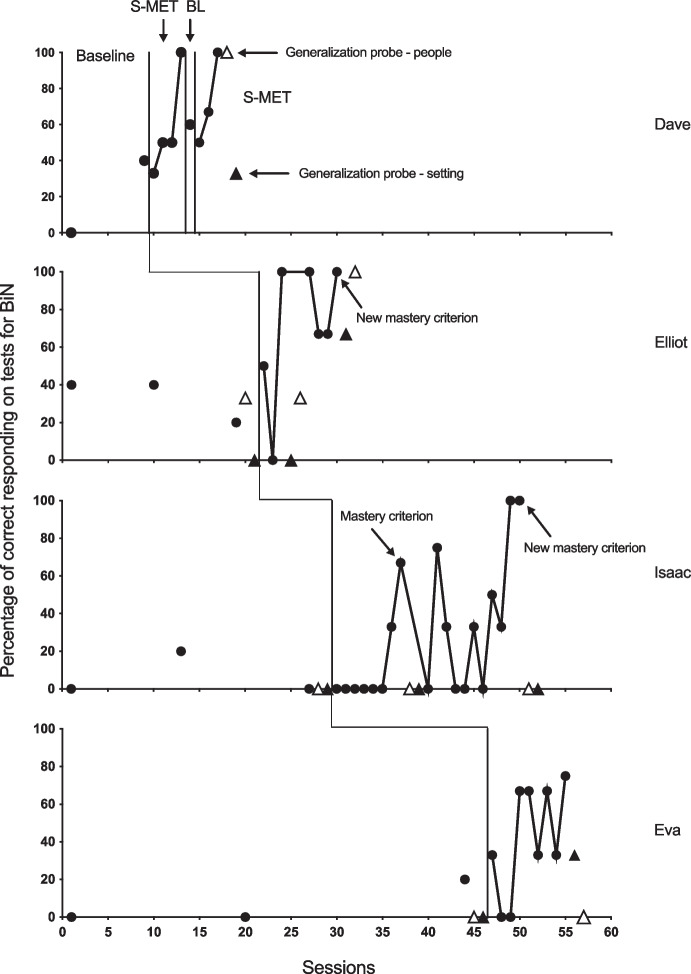
Percentage of correct responding to BiN probes in baseline and training sessions for Dave, Elliot, Isaac, and Eva. *Note.* Data during S-MET only include correct responding during BiN probes. Generalization probes are conducted as speaker probes only for Elliot, Isaac, and Eva. The mastery criterion was three consecutive correct BiN probes. The new mastery criterion was five consecutive correct BiN probes

Figure 1 shows Dave, Elliot, Isaac, and Eva’s performance during baseline probes, intervention, and generalization probes. The data show that responding for three out of four participants, Dave, Elliot, and Isaac, met the mastery criterion for BiN. All four participants showed improvement on BiN probes during the intervention.

**Fig. 2:**
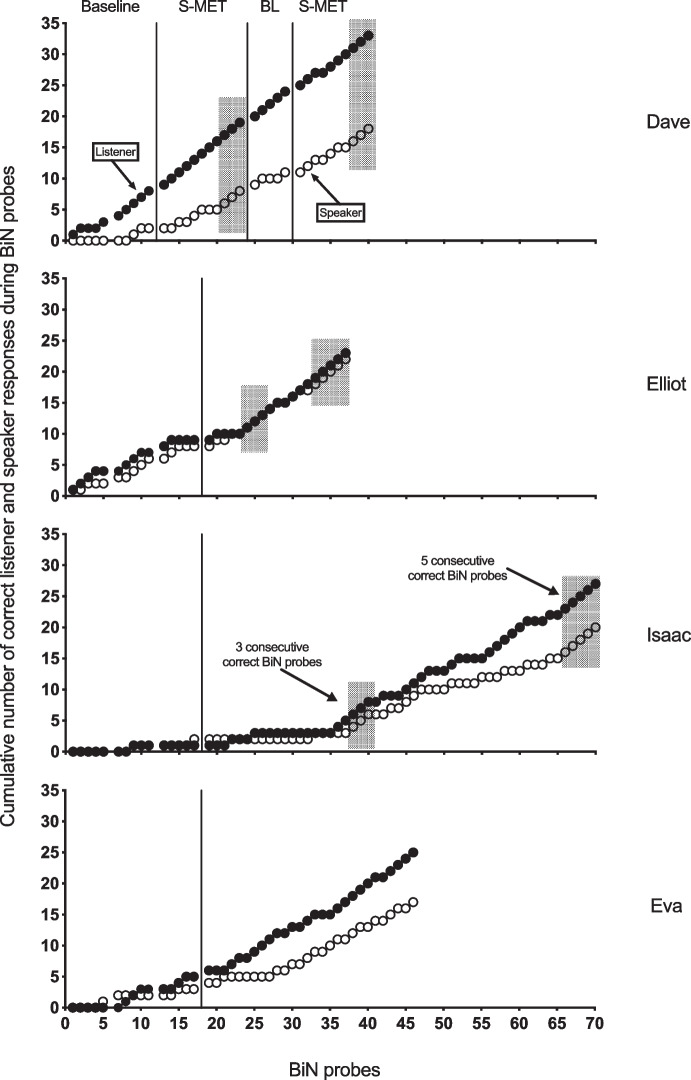
Cumulative number of mastered listener and speaker responses during BiN probes for Dave, Elliot, Isaac and Eva. *Note.* A correct listener test consists of eight correct trials. A correct speaker test consists of five correct trials

In the pre-experimental assessment, Dave echoed every stimulus but one (see Table 4). Dave did not tact any stimuli, but responded as a listener for 10 out of 51. Thus, there were 40 stimuli that were available to use during the study. Baseline sessions consisted of five BiN probes. As Fig. 1 shows, Dave’s baseline performance was not stable. In the first baseline probe session, he did not emit any correct responses. In the second session, he mastered both the listener and speaker responses for two out of the five stimuli (40%). Dave mastered eight out of 10 listener probes in baseline, indicating that he had almost mastered the listener part of BiN. It took Dave four intervention sessions, including a total of 11 stimuli for responding to meet the criterion for BiN, defined as mastering three consecutive BiN probes (see Fig. 2). Five days after responding met mastery, due to experimenter error, five new stimuli were probed in the absence of intervention, resulting in the reintroduction of the baseline condition. The result was a reduction in correct responding from 100 to 60 percent (from 5/5 to 3/5 stimuli). The intervention was reintroduced and training was conducted until Dave’s responding met mastery. This time, the criterion was reached after three sessions and 10 stimuli. Figure 1 shows that, for the post-training generalization probes, he got all three (100%) of the generalization probes correct with different people, and one out of three (33%) in other settings.

**Table 4:** Results from pre-experimental assessment and serial multiple exemplar training for each participant

Measure	Dave	Elliot	Isaac	Eva
Pre-experimental assessment				
Echoic assessment	50/51	53/53	92/95	66/83
Speaker assessment	0/51	0/53	17/95	14/83
Listener assessment	10/51	18/53	28/95	23/83
Stimuli assigned to baseline probes	10	15	15	15
Stimuli assigned to S-MET	30	20	55^a^	28
Pre-treatment generalization probes				
People	–	1/3	0/3	0/3
Settings	–	0/3	0/3	0/3
S-MET – total number of stimuli trained to mastery criterion^b^	11	8	22	27^c^
S-MET – total number of stimuli trained to new mastery criterion^d^	–	19	52	–
Post-treatment generalization probes				
People	3/3	1/3	0/3	0/3
Settings	1/3	0/3	0/3	1/3
Post-treatment generalization probes – new mastery criterion^d^				
People	–	3/3	0/3	–
Settings	–	2/3	0/3	–

Scores for pre-experimental assessment are stimuli correct/stimuli tested. Scores for generalization probes are stimuli correct/stimuli probed

^a^When a new criterion was set for Isaac, 10 new stimuli were pretested and prepared for S-MET in addition to the 45 prepared initially

^b^Three consecutive correct BiN probes

^c^The mastery criterion was not reached

^d^Five consecutive correct BiN probes

Elliot echoed all the words during the pre-experimental assessment. He did not master any speaker responses, but he could respond as a listener to 18 out of 53 stimuli. Of the remaining 35 stimuli, 15 were selected for baseline probes. Elliot had a stable but low baseline. During S-MET, Elliot’s responding reached the mastery criterion during three BiN probes after only three sessions using eight stimuli (see Fig. 2). Elliot’s responding reached the new criterion of five BiN probes within a total of seven sessions, using 19 stimuli. The generalization probes showed a marked improvement in his performance from one out of six before the intervention and, after the first mastery criterion was met, and five out of six post-intervention after responding met the new criterion.

For Isaac, a total of 95 stimuli were assessed during the pre-experimental assessment. Isaac could not echo three of the stimuli. He tacted 17, and he responded as a listener to 28 of them. Seventy stimuli remained for the baseline and treatment conditions. Isaac’s responding was low and stable during baseline. His responding during S-MET met the mastery criterion (three consecutive BiN probes) within eight sessions using 22 stimuli, and met the new mastery criterion (five consecutive BiN probes) within 19 sessions using a total of 52 stimuli (see Fig. 2). There was no generalization of BiN across people and settings.

For Eva, the pre-experimental assessment showed that she could not echo 17 of the 83 stimuli that were probed. She tacted 14 of them, and identified 23 during the listener probes. She did not tact any of the stimuli she could not echo. That left her with 43 stimuli, of which 15 were selected for baseline probes. Eva responded correctly during one BiN probe for the third of the five stimuli in the last baseline probe session before the intervention. The last baseline session had to be postponed for 11 days because she was sick. Eva’s responding did not meet the mastery criterion (three consecutive BiN probes); however, her responding improved compared to baseline (Fig. 1). In the last training session, she got three out of the four probed stimuli correct (75% correct), but not three consecutively. Unfortunately, the experiment was stopped due to a holiday break. Generalization probes were conducted in case the experiment could not be continued after the break. The experimenters became sick which prevented further training and the experiment was concluded. Generalization probes for Eva showed no correct responses prior to the intervention and one of six correct after the intervention ended. As shown in Fig. 2, Eva’s responding did not meet the mastery criterion during S-MET, but she completed eight sessions and learned to respond to 26 stimuli. See Table 4 for a summary of the results.

Cumulative data are displayed in Fig. 2. Listener and speaker functions develop across successive probed stimuli as a result of the S-MET. At the start of the training, after observing the experimenter tacting untrained stimuli as part of BiN probes, Dave usually mastered the listening part when it was probed. However, he responded to a lesser extent as a speaker without direct training. For both Isaac and Eva, training improved the listener responding of BiN before the speaker part, while, in Elliot’s case, there was no difference. For Isaac, the effect of S-MET on both listener and speaker responding was weaker compared to Dave and Elliot, as he required training with twice as many stimuli for responding to reach the mastery criterion (for comparison, see also Table 4).

## Discussion

The current study evaluated the effect of a serial multiple exemplar training (S-MET) procedure on BiN in four children with autism. The results showed that three out of the four participants’ responding reached the mastery criterion during BiN probes after S-MET. The fourth participant improved her BiN skills, but her responding did not reach the mastery criterion. After the completion of the S-MET, generalization probes across three people and three settings were conducted for all four participants. BiN in S-MET probes were above baseline; however, BiN decreased during post-training generalization probes for all four participants, suggesting that BiN was not fully established. Despite this, the increase in BiN suggests a potential emerging BiN repertoire and that S-MET may be considered a promising intervention.

Maintenance probes were not conducted in the current study. However, data indicate a lack of maintenance of BiN. Dave’s data suggest that S-MET only led to moderate maintenance since his correct responding during the BiN probes decreased after a few days without training. The unplanned withdrawal may have served as a maintenance probe. The variable responding seen during generalization probes may also be interpreted as a measure of skill maintenance since they were carried out a few hours to a day after the participants’ responding reached the BiN criterion in training. For Isaac and Eva, probe data showed close to no generalization of BiN skills, while, for Dave and Elliot, some stimulus generalization was demonstrated. Elliot’s responding only improved during generalization probes after a higher mastery criterion was introduced (three to five correct during BiN probes). For Isaac, a higher mastery criterion did not affect generalization.

A unique part of the training procedure in the current study was the manner in which the listener responses were trained. We required an echoic response from the learner during each listener trial. When we provided the instruction (e.g., “point to wasp”), the participants repeated “wasp,” before they pointed to the picture of wasp. If the participant did not repeat “wasp” prior to the pointing, we provided a prompt on the next trial to ensure that the echoic response occurred together with the pointing response. Most participants independently echoed the name of the stimulus after a few training sessions. In addition to providing an opportunity for the participants to respond as listeners, requiring the echoic response during listener trials allowed the participant to respond as both a listener and a speaker, and for the experimenter to reinforce both listener and speaker behavior during those training trials. This may have increased the likelihood that the participant responded as a speaker and emitted the tact in the presence of the stimulus during later probes. For example, by providing reinforcers for correct listener responses (hearing “wasp” and pointing to wasp) at the same time as the participant repeated “wasp,” the experimenter would probably reinforce the tact (see wasp – say “wasp”). By repeating their own echoics (overt or covert), the participants would increase the chances that these episodes would occur where the non-verbal stimulus preceded the verbal stimulus as in a tact (e.g., seeing a wasp and then saying “wasp”). In the current study, we used echoics as a prompt during the speaker trials and as a response requirement during the listener trials. There is some evidence that the frequency of a child’s echoic responding of the adult’s tacts result in improved BiN skills (Longano & Greer, 2015; Pérez-González et al., 2014).

Some research indicates that training multiple exemplars concurrently (C-MET) could result in greater stimulus or response generalization for some participants, compared to S-MET (Wunderlich et al., 2014). While both S-MET and C-MET produce generalization, for some participants C-MET seems to establish generalization earlier in training (Wunderlich et al., 2014). On the other hand, S-MET will probably have some advantages in that not all children need to learn sets of multiple stimuli. Thus, serial training may save both time, resources, and effort. During S-MET training, teachers may be able to evaluate the establishment of BiN earlier compared to C-MET and resultantly stop teaching when generalization of BiN to new exemplars occurs. As Fig. 2 shows, S-MET allowed us to see exactly when generalized responding occurred. It allowed us to stop when BiN was reached or extend the training with the sufficient number of exemplars necessary to establish BiN. As Eldevik et al. (2016) pointed out, “This can save precious teaching time and avoids unneeded repetition” (p. 497). S-MET seems to be especially suitable for monitoring generalization effects during interventions. In this study, new untrained stimuli were probed for BiN after each trained stimulus during the intervention. Probes for stimulus generalization could easily be interspersed between intervention sessions. This enables an assessment of both emergence of BiN in training (generalized responding to new stimuli) and generalization of BiN to other people and settings. Therefore, this approach allows for the mastery criterion to be individualized. S-MET may be easier for trainers to implement because of the reduced number of discriminations involved during S-MET sessions compared to concurrent training of stimulus exemplars (Schnell et al., 2018). S-MET typically consist of training one target stimulus at a time compared to sets of five stimuli trained simultaneously in MEI. Some trainers may find MEI complicated to implement because both responses (e.g., matching-to-sample, impure tacts, pure tacts, and point-to responses) and sets of usually five training stimuli have to be rotated for each trial. This may be impractical to do in a clinical setting and require more planning and guidance of staff compared to S-MET. Another advantage of S-MET compared to MEI is that it may reduce extended probing under extinction conditions. BiN probe sessions conducted in MEI could entail up to 60 consecutive trials of probing under extinction conditions (Olaff et al., 2017). In the current study, only 13 consecutive trials were conducted.

There are several differences in this study compared to BiN-studies using MEI, as reported in Gilic and Greer (2011) and Greer et al. (2007). This makes it difficult to directly compare the results. First, the procedure for probing in BiN is different. In the current study, a pairing naming procedure similar to that described by Carnerero and Pérez-González (2014) was used. The BiN probing procedure we used in the current study did not include the matching to sample training that Greer and colleagues (Greer et al., 2005) described, nor the uninstructed speaker response probe (pure tact) after the observation part. The BiN probes used in the current study may lead to improved experimental control since they involve less exposure to the stimulus material. In some of the MEI studies, the initial training phases could be a confound since repeated identity matching trials in BiN probes, together with the adult tacting, could make it difficult to isolate the effects of MEI from the effect of the naming probes prior to MEI (Olaff et al., 2017). For some children, repeated exposure to the stimulus material has led to improved performance in subsequent listener and speaker probes (Carnerero & Pérez-González, 2014; Rosales et al., 2012). In previous studies evaluating procedures for establishing BiN, single case experimental designs with pre- and post-testing have been used (Gilic & Greer, 2011; Greer et al., 2005, 2007; Hawkins et al., 2009). In the current study, efforts were made to increase experimental control by including repeated baseline probes.

Greer et al. (2007) compared the use of single exemplar instruction (SEI) to MEI in relation to establishing BiN. S-MET differs from SEI in that, in S-MET, you only teach one exemplar to mastery at a time, first the listener relation, then the tact relation, both in the same session, but separately. On the other hand, Greer et al. (2007) described that in SEI they: “...taught all topographies separately in massed 20-learn unit [learn unit] sessions.” (p. 118). Then, “...we conducted single exemplar instruction for second set using massed learn unit session with match only first, then the point-to, followed by pure tact, and then impure tact responses, respectively.” (Greer et al., 2007, p. 119). Serial multiple exemplar training differs from both MEI or SEI, but is probably more similar to SEI because the responses are not rotated between each trial, but are taught separately.

There was a great deal of variation between participants in the study in terms of correct responding during training. Dave and Elliot performed better than Isaac and required training with fewer stimuli before the BiN mastery criterion was reached. Isaac required training with 22 stimuli before responding met the mastery criterion, compared to Dave’s 11 stimuli and Elliot’s eight. Eva’s responding did not reach the mastery criterion, and when the training had to be stopped due to a holiday break and sickness, training had been conducted with 26 stimuli. The participants’ ABLLS-R scores (Table 1) showed that there were clear differences in scores between participants on skills critical to BiN, such as listening skills (receptive language) and speaking skills (tacting). Dave and Elliot mastered 89% and 93% of the listening skills, respectively, compared to 73% and 60% for Isaac and Eva. The differences were about the same for speaker skills. The marked differences in skill profile between the participants could also explain the individual variation seen in the response to S-MET. Isaac, in particular, but also Eva, initially showed little response to the intervention. Isaac started with six sessions without any correct probes before responding reached the mastery criterion. After that, his responding is also characterized by great variability from 0% to 80% mastery during training sessions. Some of the same variability was seen in Eva’s responding. For participants with fewer listener and speaker skills, like Isaac and Eva, days without training (weekends, etc.) may have negatively impacted their responding during BiN probes.

The behavioral processes that underlie the generative nature of BiN can be conceptualized in multiple ways (Stewart et al., 2013). Some scholars have considered BiN to be a form of derived relational responding (Hayes et al., 2001; Ming et al., 2014). According to Relational Frame Theory, BiN can be understood as one of several relational frames, where responding can be seen as reflecting coordination or a bidirectional relation between an object and the related word (Stewart et al., 2013). A history of multiple exemplar experiences across word–object and object–word relations and contextual cues, such as “that is” and “name of,” helps establish the symmetric or bidirectional pattern known in BiN (Stewart et al., 2013).

The current study probably resembles how natural learning occurs in children. First, the pairing procedure consists of observing others naming stimuli without a response requirement. This is how Horne and Lowe (1996) describe a naming experience when the caregiver simultaneously “points to” and “utters the name” of a stimulus for a child. Second, an S-MET strategy may be very similar to how language learning takes place in natural settings. Dyadic interactions between caregivers and the child, typically in the form of three-term contingencies, are prominent in early language acquisition (Moerk, 1983). Eldevik et al. (2016) described – in the context of teaching past-tense verbs – how S-MET could be reminiscent of how children learn one verb in one situation, and the next in another situation, until the generalized use of past-tense verbs is established. This could also be the case for other language skills, such as BiN.

Non-concurrent multiple baseline designs (NCBD) offer a flexible approach because they make it possible to start baseline measurements at different times with different participants. It is therefore not necessary to recruit several participants at the same time who need the same type of intervention (Christ, 2007). Although there is some debate about the degree of experimental control that can be achieved with this design (Christ, 2007; Carr, 2005; Novotny et al., 2014), there is nonetheless agreement that the NCBD is more vulnerable to unwanted influences from extraneous events on the dependent variable (history effects), than with a concurrent multiple baseline design. According to Carr (2005), some of the challenges with NCBD can be counteracted by introducing a short withdrawal in one of the baselines since it is verification of the intervention effect that is achieved to a lesser extent with NCBD.

There are several potential limitations with this experiment. First, Dave’s, and to a lesser degree Eva’s, responding during baseline showed an upward trend. Despite Dave showing an upward trend in baseline, his response pattern was variable, and his responding had not met the mastery criterion for BiN. We initiated the intervention to see whether his responding could meet the mastery criterion and display a more stable response pattern. Dave’s trending baseline reduces the possibility of interpreting the results because it could be that he was engaging in BiN. Although the reduction in correct responding during the accidental BiN probes without intervention appears to show a functional relationship with S-MET, Dave’s responding during baseline could represent a potential limitation. Eva’s responding during baseline showed a slight upward trend from zero during the first two probe sessions to one out of five (20%) in the final probe session before the intervention. The baseline probes for Eva were conducted over a period of 44 days, and the treatment from start to finish only lasted 11 days. Even though her performance in the intervention conditions showed improvement compared to baseline, her baseline performance makes it harder to rule out the possibility that she was starting to engage in BiN before the intervention was introduced. Eva’s baseline was scheduled to be 35 days. When illness prolonged the time to the last probe, this reduced the possibility of knowing whether her response pattern was partly a function of maturation. In future studies, replications with participants with more stable baseline performances are required. Using a concurrent multiple baseline design will allow the researcher to extend the baseline to try to achieve more stable responding before the intervention (Christ, 2007).

A second potential limitation is the way in which the generalization probes for Elliot, Isaac, and Eva were conducted. We measured only the speaker part of BiN. Just probing the speaker part of the skill set will not be a complete measurement of BiN since it consists of both listener and speaker responses. This means that the data from generalization probes must be interpreted with caution. Future studies need to utilize generalization probes where listener responses are also measured in order to understand the extent to which BiN generalizes across conditions.

A third potential limitation is the assessment of the bidirectional naming repertoire in the BiN probes, where one could never be completely sure that the skills were not just measured from one direction (tact–listener). When the experimenter named a new stimulus, the participant’s echoic behavior was potentially evoked, and for some the tact may be established. There is a possibility of the tact being trained, even though the instructional agent does not prompt or deliver reinforcement for the participant’s responding. In the experiment, the probe of the tact took place a few minutes after this episode and could therefore be a probe of tact training. A possible solution could be to explicitly train the listener part of a stimulus (by the adult naming a stimulus and prompting the participant to touch or show him/her the correct one), and probe speaking responses (listener–tact) to ensure that the probe is an assessment of the bidirectionality of the skill.

A fourth potential limitation of this study was that the listener probe was completed before the speaker probe for each stimulus. This provided the participants with the verbal stimulus of the target stimulus at three times before asking for them. Future research should reverse the order of probes so that probes for speaker responding are being conducted before probes for listener responding.

Finally, a fifth possible limitation was the use of known distractors while probing listener responding. Known distractors could increase the likelihood of the participants learning through exclusion.

Future research should identify the prerequisite skills important to acquiring BiN. The participants show some variation in listener and speaker skills and this may have accounted for the results in this study. In future studies, it could be useful to divide participants into groups where key skills (e.g., speaking and listening skills) are more in line with each other to be able to predict and select participants who will benefit from the intervention. Future investigations should also include maintenance probes to better understand the extent to which skills may endure after the intervention.

The current study evaluated the effects of a serial multiple exemplar training procedure on the development of BiN in preschool children with autism. Despite some limitations, the results are promising and warrant further research. Experiments that endeavor to clarify the basic processes of how children acquire bidirectional naming could contribute to both a greater understanding of determinants of language development and the development of effective procedures for use in applied settings. There are several potential benefits of acquiring BiN (Greer & Longano, 2010). Procedures that help children with autism learn to engage in BiN would contribute to reducing some resource-intensive features related to EIBI programming, and more importantly, would equip these learners with the relevant verbal behavior to maximize their potential to acquire important communication and verbal behavior skills.

## Data Availability

Data are available upon request to the first author.

## References

[CR1] Carnerero JJ, Pérez-González LA (2014). Induction of naming after observing visual stimuli and their names in children with autism. Research in Developmental Disabilities.

[CR2] Carr JE (2005). Recommendations for reporting multiple-baseline designs across participants. Behavioral Interventions.

[CR3] Christ TJ (2007). Experimental control and threats to internal validity of concurrent and nonconcurrent multiple baseline designs. Psychology in the Schools.

[CR4] Eikeseth S, Nesset R (2003). Behavioral treatment of children with phonological disorder: The efficacy of vocal imitation and sufficient-response-exemplar training. Journal of Applied Behavior Analysis.

[CR5] Eldevik, S., Kazemi, E., & Elsky, E. (2016). Generalized use of past tense verbs in children with autism following a sufficient exemplar training procedure. *International Electronic Journal of Elementary Education,**9*(2), 485–498. Retrieved from https://www.iejee.com/index.php/IEJEE/article/view/171.

[CR6] Fiorile CA, Greer RD (2007). The induction of naming in children with no prior tact responses as a function of multiple exemplar histories of instruction. The Analysis of Verbal Behavior.

[CR7] Gilic L, Greer RD (2011). Establishing naming in typically developing two-year-old children as a function of multiple exemplar speaker and listener experiences. The Analysis of Verbal Behavior.

[CR8] Greer RD, Longano J (2010). A rose by naming: How we may learn how to do it. The Analysis of Verbal Behavior.

[CR9] Greer RD, Ross DE (2008). Verbal behavior analysis: Inducing and expanding new verbal capabilities in children with language delays.

[CR10] Greer RD, Stolfi L, Chavez-Brown M, Rivera-Valdes C (2005). The emergence of the listener to speaker component of naming in children as a function of multiple exemplar instruction. The Analysis of Verbal Behavior.

[CR11] Greer RD, Stolfi L, Pistoljevic N (2007). Emergence of naming in preschoolers: A comparison of multiple and single exemplar instruction. European Journal Of Behavior Analysis.

[CR12] Hawkins E, Kingsdorf S, Charnock J, Szabo M, Gautreaux G (2009). Effects of multiple exemplar instruction on naming. European Journal Of Behavior Analysis.

[CR13] Hayes SC, Fox E, Gifford EV, Wilson KG, Hayes SC, Barnes-Holmes D, Roche B (2001). Derived relational respondings as learned behavior. Relational Frame Theory. A Post-Skinnerian Account of Human Language and Cognition.

[CR14] Horne PJ, Lowe CF (1996). On the origins of naming and other symbolic behavior. Journal of the Experimental Analysis of Behavior.

[CR15] Jahr E (2001). Teaching children with autism to answer novel wh-questions by utilizing a multiple exemplar strategy. Research in Developmental Disabilities.

[CR16] Jahr E, Eldevik S, Eikeseth S (2000). Teaching children with autism to initiate and sustain cooperative play. Research in Developmental Disabilities.

[CR17] Labrell F, Geert PV, Declercq C, Baltazart V, Caillies S, Olivier M, Sourn-Bissaoui SL (2014). ‘Speaking volumes’: A longitudinal study of lexical and grammatical growth between 17 and 42 months. First Language.

[CR18] LaFrance DL, Miguel CF, Tarbox J, Dixon D, Sturmey P, Matson J (2014). Teaching Verbal behavior to children with autism spectrum disorders. Handbook of Early Intervention for Autism Spectrum Disorders.

[CR19] LaFrance DL, Tarbox J (2020). The importance of multiple exemplar instruction in the establishment of novel verbal behavior. Journal of Applied Behavior Analysis.

[CR20] Lee GP, Miguel CF, Darcey EK, Jennings AM (2015). A further evaluation of the effects of listener training on derived categorization and speaker behavior in children with autism. Research in Autism Spectrum Disorders.

[CR21] Longano JM, Greer RD (2015). Is the source of reinforcement for naming multiple conditioned reinforcers for observing responses?. The Analysis of Verbal Behavior.

[CR22] Lord C, Rutter M, DiLavore PC, Risi S, Gotham K, Bishop S (2012). Autism Diagnostic Observation Schedule, Second Edition (ADOS-2) Manual (Part I): Modules 1–4.

[CR23] Lowe CF, Horne PJ, Hughes JC (2005). Naming and categorization in young children III. Vocal tact training and transfer of function. Journal of the Experimental Analysis of Behavior.

[CR24] Miguel CF (2016). Common and intraverbal bidirectional naming. The Analysis of Verbal Behavior.

[CR25] Miguel CF, Petursdottir AI, Carr JE, Michael J (2008). The role of naming in stimulus categorization by preschool children. Journal of the Experimental Analysis of Behavior.

[CR26] Ming S, Moran L, Stewart I (2014). Derived relational responding and generative language: Applications and future Directions for teaching individuals with autism spectrum disorders. European Journal Of Behavior Analysis.

[CR27] Moerk EL (1983). A behavioral analysis of controversial topics in first language acquisition: Reinforcements, corrections, modeling, input frequencies, and the three-term contingency pattern. Journal of Psycholinguistic Research.

[CR28] Novotny MA, Sharp KJ, Rapp JT, Jelinski JD, Lood EA, Steffes AK, Ma M (2014). False positives with visual analysis for nonconcurrent multiple baseline designs and ABAB designs: Preliminary findings. Research in Autism Spectrum Disorders.

[CR29] Olaff HS, Holth P (2020). The Emergence of Bidirectional Naming Through Sequential Operant Instruction Following the Establishment of Conditioned Social Reinforcers. The Analysis of Verbal Behavior.

[CR30] Olaff HS, Ona HN, Holth P (2017). Establishment of naming in children with autism through multiple response-exemplar training. Behavioral Development Bulletin.

[CR31] Partington JW (2006). The Assessment of Basic Language and Learning Skills-Revised (ABLLS-R): An assessment, curriculum guide and skills tracking system for children with autism or other developmental disabilities.

[CR32] Pérez-González LA, Cereijo-Blanco N, Carnerero JJ (2014). Emerging tacts and selections from previous learned skills: A comparison between two types of naming. The Analysis of Verbal Behavior.

[CR33] Rosales R, Rehfeldt RA, Huffman N (2012). Examining the utility of the stimulus pairing observation procedure with preschool children learning a second language. Journal of Applied Behavior Analysis.

[CR34] Rutter M, Le Couteur A, Lord C (2003). ADI-R Autism Diagnostic Interview Revised. Manual.

[CR35] Schnell LK, Vladescu JC, Kodak T, Nottingham CL (2018). Comparing procedures on the acquisition and generalization of tacts for children with autism spectrum disorder. Journal of Applied Behavior Analysis.

[CR36] Stewart I, McElwee J, Ming S (2013). Language generativity, response generalization, and derived relational responding. The Analysis of Verbal Behavior.

[CR37] Watson PJ, Workman EA (1981). The non-concurrent baseline across-individuals design: An extension of the traditional multiple baseline design. Journal of behavior therapy and experimental psychiatry.

[CR38] White, R. O., Liberty, K. A., Haring, N. G., Billingsley, F. F., Boer, M., Burrage, A., ..., Sessoms, I. (1998). Review and analysis of strategies for generalization. In N. G. Haring (Ed.), *Generalization for Students with Severe Handicaps: Strategies and Solutions* (pp. 15–45). University Washington Press.

[CR39] World Health Organization (2007). ICD-10: Psykiske lidelser og atferdsforstyrrelser: Kliniske beskrivelser og diagnostiske retningslinjer.

[CR40] Wójcik M, Eikeseth S, Eldevik S, Budzińska A (2020). Teaching children with autism to request items using audio scripts, interrupted chain procedure and sufficient exemplar training. Behavioral Interventions.

[CR41] Wunderlich KL, Vollmer TR, Donaldson JM, Phillips CL (2014). Effects of serial and concurrent training on acquisition and generalization. Journal of Applied Behavior Analysis.

